# Affect Reactivity to Daily Stressors Mediates the Relationship Between Personality Traits and Physical Health

**DOI:** 10.1093/geroni/igab046.964

**Published:** 2021-12-17

**Authors:** Kate Leger, Nicholas Turiano, William Bowling, Jessica Burris, David Almeida

**Affiliations:** 1 University of Kentucky, Lexington, Kentucky, United States; 2 West Virginia University, Morgantown, West Virginia, United States; 3 Pennsylvania State University, University Park, Pennsylvania, United States

## Abstract

Researchers hypothesize that how people react to daily stressful events partly explains the personality-health relationship, yet no study has examined longitudinal associations between these factors. The current study examined the role of negative affect reactivity to daily stressors as a mediating pathway between personality and physical health outcomes using three waves of data spanning 20-years from a nationwide probability sample of 1,176 adults. Results indicate that Wave 1 neuroticism was associated with greater negative affect reactivity at Wave 2, which then predicted the development of chronic conditions and functional limitations at Wave 3. Higher conscientiousness was associated with less negative affect reactivity, which in turn predicted better physical health at Wave 3. Negative affect reactivity partially mediated both personality traits and physical. These findings highlight the usefulness of using a daily stress framework for understanding how personality impacts health over time, which has important implications for disease prevention.

